# Mini-Batch Alignment: A Deep-Learning Model for Domain Factor-Independent Feature Extraction for Wi-Fi–CSI Data †

**DOI:** 10.3390/s23239534

**Published:** 2023-11-30

**Authors:** Bram van Berlo, Camiel Oerlemans, Francesca Luigia Marogna, Tanir Ozcelebi, Nirvana Meratnia

**Affiliations:** Department of Mathematics and Computer Science, Eindhoven University of Technology, P.O. Box 513, 5600 MB Eindhoven, The Netherlandsfrancescamarogna.fm@gmail.com (F.L.M.); t.ozcelebi@tue.nl (T.O.); n.meratnia@tue.nl (N.M.)

**Keywords:** device-free sensing, unobtrusive sensing, Wi-Fi–CSI, domain shift, domain-independent learning, domain adaptation

## Abstract

Unobtrusive sensing (device-free sensing) aims to embed sensing into our daily lives. This is achievable by re-purposing communication technologies already used in our environments. Wireless Fidelity (Wi-Fi) sensing, using Channel State Information (CSI) measurement data, seems to be a perfect fit for this purpose since Wi-Fi networks are already omnipresent. However, a big challenge in this regard is CSI data being sensitive to ‘domain factors’ such as the position and orientation of a subject performing an activity or gesture. Due to these factors, CSI signal disturbances vary, causing domain shifts. Shifts lead to the lack of inference generalization, i.e., the model does not always perform well on unseen data during testing. We present a domain factor-independent feature-extraction pipeline called ‘mini-batch alignment’. Mini-batch alignment steers a feature-extraction model’s training process such that it is unable to separate intermediate feature-probability density functions of input data batches seen previously from the current input data batch. By means of this steering technique, we hypothesize that mini-batch alignment (i) absolves the need for providing a domain label, (ii) reduces pipeline re-building and re-training likelihood when encountering latent domain factors, and (iii) absolves the need for extra model storage and training time. We test this hypothesis via a vast number of performance-evaluation experiments. The experiments involve both one- and two-domain-factor leave-out cross-validation, two open-source gesture-recognition datasets called SignFi and Widar3, two pre-processed input types called Doppler Frequency Spectrum (DFS) and Gramian Angular Difference Field (GADF), and several existing domain-shift mitigation techniques. We show that mini-batch alignment performs on a par with other domain-shift mitigation techniques in both position and orientation one-domain leave-out cross-validation using the Widar3 dataset and DFS as input type. When considering a memory-complexity-reduced version of the GADF as input type, mini-batch alignment shows hints of recuperating performance regarding a standard baseline model to the extent that no additional performance due to weight steering is lost in both one-domain-factor leave-out and two-orientation-domain-factor leave-out cross-validation scenarios. However, this is not enough evidence that the mini-batch alignment hypothesis is valid. We identified pitfalls leading up to the hypothesis invalidation: (i) lack of good-quality benchmark datasets, (ii) invalid probability distribution assumptions, and (iii) non-linear distribution scaling issues.

## 1. Introduction

Human gesture-recognition and activity-monitoring technologies are widely used in numerous applications such as virtual games, self-driving cars, smart homes, and security surveillance. The main issue of currently used human gesture and activity-recognition approaches is the fact that they use sensing devices such as (depth) cameras [1,2,3] or wearable devices [4,5]. Wearable devices are not ideal, as they are not only inconvenient due to requiring the user to always carry a physical device on them, but can also be extremely expensive [6]. Camera-based human activity-recognition devices pose many concerns for users as there is always a lingering risk of privacy leakage [7].

Because of these limitations, active research is being conducted with radio-based unobtrusive sensing through monitoring objects, individuals, or crowds by collecting radio data in their environment. The motivation behind unobtrusive radio-based sensing is that the presence, movement, activities, and behavior of entities such as humans change the radio signal propagation through the environment. It is possible to link these changes to the sources causing these changes, and as such, they can facilitate activity/behavior recognition. We focus on a specific type of radio-based unobtrusive sensing called Wireless Fidelity–Channel State Information (Wi-Fi–CSI) sensing [8]. Wi-Fi has been the ideal solution as it is low-cost and easy to deploy because communication hardware, such as specific Network Interface Cards (NICs), can be found in most households. Moreover, it has the advantages of being device-free, non-intrusive, and privacy-friendly (in contrast to video and audio).

CSI is an informative propagation metric of Wi-Fi communication signals [9]. CSI measurements capture how signals travel across time and in a specific space. CSI is represented via complex numbers denoting the amplitude attenuation and phase shift of a signal being propagated from a transmitter antenna to a receiver antenna at certain subcarrier frequencies in a carrier frequency band along multiple paths [6]. Generally speaking, activity recognition using CSI data works as follows: when a person between a transmitting and receiving antenna performs an activity/gesture, e.g., clapping or waving, the communication signal is disturbed due to reflections and interference of the signal. This is again reflected in the observed amplitude attenuation and phase shift in CSI data.

Researchers have developed various machine-learning solutions to recognize or classify different gestures or activities from Wi-Fi–CSI data. The main difference between gesture and activity recognition is related to activity granularity. The amount of frame-to-frame signal propagation variations observed in the radio data due to an activity/gesture (e.g., when moving an arm from point A to point B) is what defines activity granularity. ‘Coarse-grained’ activities, e.g., walking and jumping, are those for which people use their whole body [10,11]. Activities can also be ‘fine-grained’. In this case, there is less potential for signals disturbed by the aforementioned activities to encounter vastly different propagation effects such as absorption or deflection (pushing, pulling, or sign language) [12,13,14,15,16]. Researchers used ‘conventional’ machine-learning algorithms such as K-Nearest Neighbors [17] and Support Vector Machines (SVMs) [16], but also deep-learning [10,11,13,15] algorithms to recognize a gesture by analyzing increasingly complex feature representations extracted from pre-processed CSI data via sub-model chaining. In this paper, we focus on deep-learning algorithms.

### Problem Statement and Contributions

Ideally, data used for training deep-learning models are influenced solely by the phenomena that determine the output of the end classification task (e.g., classifying gestures, activities, etc.). However, in the case of Wi-Fi–CSI data collection in real life, data are influenced by domain factors, which include but are not limited to the physical characteristics of an environment in which the activity/gesture is performed, the physical characteristics of the individual, and the location and orientation of the sensing system. Domain factor variations lead to task output inconsistencies since the features collected from the same gesture may have a very different structural representation. These inconsistencies collectively lead to ‘domain shifts’. Domain shifts are changes in the distribution of data features in the datasets used for training and testing deep-learning models. This issue leads to a lack of generalization ability of the deep-learning model employed to perform the recognition task [8]. To create more robust and less domain-dependent models, different methods such as attention-based learning [12], few-shot learning with neural networks [14], reconstruction [9], and domain label classification [11] have been used.

The work presented in this paper is motivated by the fact that the use of a domain label for steering a model’s training process requires re-building and re-training the model from scratch every time a new domain is encountered [13]. The attention scheme presented in [18] also suffers from this problem because it assumes the existence of a fixed number of domains. The use of input reconstruction as an additional task requires two almost identical additional deep-learning submodels, i.e., an encoder and a decoder [9], resulting in storage and training time overhead. To address this, we defined and evaluated the following hypothesis: *domain factor independence and adaptation limitations can be absolved by steering a feature-extraction model’s training process in such a way that the model loses the ability to separate intermediate feature-probability density functions (PDFs) of input data batches seen previously from the current input data batch PDF artificially permuted with a diffeomorphism* (a smooth, differentiable, and invertible element-wise map function which is used to alter the PDF shape). To validate this hypothesis, we propose a domain-factor-independent feature-extraction pipeline called *mini-batch alignment*. Our contributions include:

- Vast mini-batch alignment classification performance evaluations with (i) open-source gesture-recognition benchmark Wi-Fi–CSI datasets [13,15], (ii) different pre-processed deep neural network input types, and (iii) different domain-shift mitigation techniques. The evaluations first indicate that mini-batch alignment performs on par with other domain-shift mitigation techniques in both position and orientation one-domain leave-out cross-validation using the Widar3 benchmark dataset and DFS input type. Second, they indicate that when considering a memory-complexity-reduced version of GADF as input type, mini-batch alignment shows hints of being able to recuperate performance regarding a standard baseline model to the extent that no additional performance due to weight steering is lost in both one-domain-factor leave-out and orientation two-domain-factor leave-out cross-validation scenarios.
- Via cross-comparison of our results with results reported in [12,13], we show that the use of specific newer backbone neural networks improves inference performance.
- We present circumstances under which mini-batch alignment and its underlying hypothesis are invalid. Additionally, We identify a set of pitfalls contributing to this. We also present new research paths for testing data feature distribution shift effect removal improvements to address them.

## 2. Wi-Fi Channel State Information

Two communication parallelization technologies that have helped Wi-Fi to cope well with an increasing number of devices and increasing communication bandwidth/data throughput requirements are called Orthogonal Frequency-Division Multiplexing (OFDM) and Multiple-Input and Multiple-Output (MIMO) [19,20]. OFDM allows the use of multiple partly overlapping subcarrier frequencies on a single communication channel, which can be used to encode and subsequently communicate a bit stream. Under the assumption that signals modulated in those subcarrier frequencies are orthogonal to each other, the signals resulting from the modulation procedure are communicated over a channel and arrive, due to natural phenomena, in the form of a single summed signal at a receiver [19]. MIMO, in the context of Wi-Fi communication, uses multiple transmitters and receivers to create multiple parallel communication channels that use the same carrier frequency band. It exploits the phenomenon that, even though the carrier frequency band is the same, signals almost never arrive at receivers alongside the same propagation path. Under the assumption that a Network Interface Controller (NIC) provides access to accurate CSI, and the propagation signatures present in CSI sufficiently differ from each other, multiple signals communicated over multiple channels can be separated [20].

CSI data extracted from an NIC is typically structured in a 4D tensor, denoted by *H*, expressed in Equation (1). Symbols i,j,kandt denote transmitter, receiver, subcarrier, and time instant. Every element in the 4D tensor represents a multi-path frequency response (i.e., propagation signature) in which $a_{p}\left(t\right)$ denotes an amplitude attenuation factor and $d_{i,j,p}$ a path length between transmitter *i*, receiver *j*, and path *p*. The path length influences the time delay, after which a receiver receives a signal. The time delay causes a receiver signal’s phase to shift compared to the transmitted signal. The symbols $f_{k}$ and *c* denote *k*th subcarrier frequency and the speed of light. Signals may arrive alongside multiple paths due to, for example, signal scattering or diffraction [21]. In addition to separating multiple communication channels with a similar carrier frequency band, CSI data can also be used to measure phenomena of interest that naturally interact with Wi-Fi signals (e.g., objects, individuals, or crowds). More information on the use of Wi-Fi–CSI in sensing applications, including a more detailed Wi-Fi–CSI data structure explanation, can be found in a survey paper written by Ma et al. [8].

(1) $$\begin{array}{c} H\in C^{\left(I\times J\times K\times T\right)}\\ H_{i,j,k,t}=\sum_{p}^{P}a_{p}\left(t\right)e^{-j2\pi d_{i,j,p}f_{k}/c} \end{array}$$

## 3. Mini-Batch Alignment

**Figure 1:**
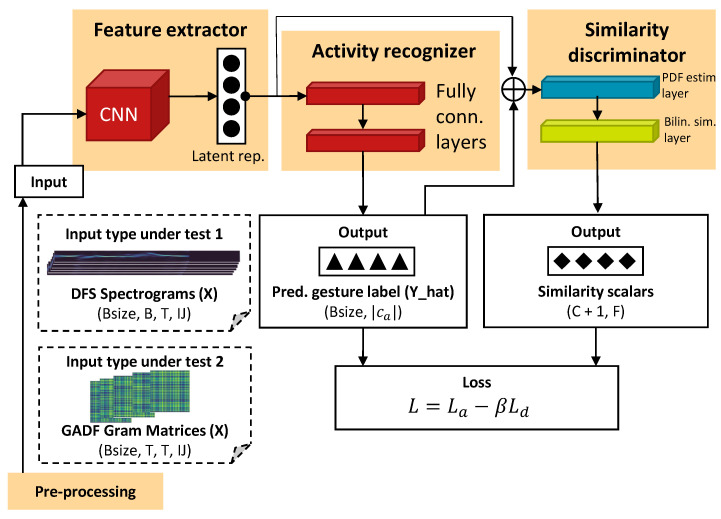
Mini-batch alignment pipeline overview. The pipeline is trained via an overall adversarial loss, which the model uses to minimize a task-specific loss while maximizing a similarity loss in parallel. The model weights are therefore steered such that the model loses the ability to separate probability density functions belonging to mini-batches seen previously from a probability density function belonging to the current mini-batch, which is artificially permuted with a diffeomorphism. The symbol $c_{a}$ denotes gesture classes and *C* the probability density function bank capacity. For more in-depth information, see Section 3.

The mini-batch alignment technique, used in the mini-batch alignment pipeline that can be found in Figure 1, forgets differences between previous PDFs of mini-batch distributions and permuted PDFs of mini-batch distributions in an iterative way. In the subsections that follow, we explain different components of the mini-batch pipeline.

Our mini-batch alignment technique is inspired by the MMD [22] loss and specific use of the Wasserstein distance [23]. The reason for this is that both techniques also involve a form of probability density estimation as an intermediate computation procedure. The mini-batch alignment technique differs from these techniques. It does not involve source and target distribution distance reduction in its loss definition and does not involve sample generation based on distance from an original distribution. The MMD loss, when minimized, brings together hidden input sample source and target distributions via empirical estimation. Empirical estimation is based on kernel feature mapping input samples to a Reproducing Kernel Hilbert Space (RKHS), computing feature means across all mapped input samples separately for the source and target distributions, and computing feature mean distance via subtraction. The specific use of the Wasserstein distance involves learning data augmentation to generate input samples with a probability distribution that is at least ρ away from the initial probability distribution [24].

### 3.1. Data Pre-Processing

Data pre-processing takes Wi-Fi–CSI data in as $R^{\left(A\times K\times T\right)}$, where *A* denotes transceiver link antenna. These data are initially reduced from a given number of transceiver link antennas in the *A* axis to 2 such that the first antenna matrix contains far greater amplitudes than the second antenna matrix, therefore adhering to an amplitude requirement for extracting Doppler shifts [25]. Amplitude adjustments are made to both antenna matrices such that the Doppler shift information in the conjugate multiplication output has a much higher amplitude [26]. The conjugate multiplication between antenna matrices is used to remove random phase offsets. This is possible because the antennas are connected to the same radio frequency oscillator. After conjugate multiplication, static and high-frequency noise components are filtered out with a low and high-pass Butterworth filter, respectively. The filters are 6th and 3rd order, respectively, and use a critical frequency of 60/500 and 2/500 half-cycles per sample. Using Principal Component Analysis (PCA), the Butterworth filter output is reduced to the first principal component.

We consider two different data input types, i.e., Doppler Frequency Shift (DFS) and Gramian Angular Difference Field (GADF) [13,27] applied to the Wi-Fi–CSI’s amplitude value. According to Wang and Oates [27], GADF is another visual way to understand time series. As time increases, corresponding values warp among different angular points on spanning circles, like water rippling. GADF preserves temporal dependency, and it contains temporal correlations [27]. DFS refers to the change in frequency of a wave caused by the relative motion between the wave source and the observer. In other words, GADF can be considered signal analysis in the time dimension, while DFS can be considered signal analysis in the frequency dimension across time. The reason we have considered both time and time-frequency dimension analysis is that time dimension analysis may provide the necessary Wi-Fi–CSI value variation in situations in which micro-Doppler components are obfuscated by dominant Doppler components. However, in dominant Non-Line-Of-Sight (NLOS) scenarios (LOS to receiver antenna is blocked, and there are no additional antennas at locations to alleviate this), receiver signal amplitude variation follows a Rayleigh fading distribution [28]. The observed Rayleigh distribution may have a high peak concentrated around a specific amplitude value without observed noteworthy tails. Therefore, Wi-Fi–CSI amplitude value variation may be quite low while there is a large noticeable difference in observed frequency components.

To acquire DFS, we transform the first principal component of the Butterworth filter output into a matrix structure $\in R^{\left(B\times T\right)}$ by Short-Time Fourier Transform (STFT). This transformation has been taken from the Widar3 [13] research project. The matrix structure represents energy (i.e., magnitude) distribution information over both a Doppler frequency bin and time dimension. The STFT output is zero-padded at the end and in the direction of the *T* axis to 2000 instants. STFT outputs for the same gesture and repetition under the same unique combination of domain factors but belonging to different transceiver links are depth-stacked together to form the input modality $x\in R^{\left(B\times T\times IJ\right)}$, in which *B* denotes the Doppler frequency bin and IJ the transceiver link dimension. Data are served to the feature extractor block in the form of mini-batches $\in R^{\left(Bsize\times B\times T\times IJ\right)}$ with size Bsize.

To acquire GADF, we convert the first principal component of the Butterworth filter output’s complex numbers into real numbers by taking the amplitude to reduce computational complexity and storage requirements. The caveat is that this leads to a reduction in performance. Afterward, the principal components amplitude vector is downsampled in the time dimension via Piecewise Aggregate Approximation (PAA) [29]. PAA involves splitting the principal component’s amplitude vector into windows and computing the mean value across the windows. The mean values are subsequently connected. Then, the PAA output is scaled to the interval [−1,1], followed-up by converting the scaled PAA output into the quasi-Gramian matrix GADF using Equation (2). The symbol ψ denotes the value scaled PAA output and *n* its vector size. When comparing the size of a GADF and DFS matrix, we notice that GADF $\in R^{\left(T,T\right)}$ is much larger than DFS $\in R^{\left(B,T\right)}$ since the amount of Doppler frequency bins is limited. Therefore, the GADF matrix is downsampled with a factor q to $R^{\left(T/q,T/q\right)}$ using bilinear interpolation. As a result, overall DFS and GADF matrix sizes are the same, and therefore, deep-learning models under test have an equal amount of computational complexity during training. During model training, we did not have enough storage disk capacity for storing the datasets in GADF form without considering down-sampling. Down-sampling with bilinear interpolation first involves computing a width and height size difference ratio. Second, for every difference ratio scaled coordinate in the downsampled image, the weighted sum between 4 neighboring matrix index coordinates in the original is computed. GADF matrices for the same gesture and repetition under the same unique combination of domain factors but belonging to different transceiver links will then be depth-stacked together to form the input modality $x\in R^{\left(T\times T\times IJ\right)}$. Data are fed to the feature extractor block in the form of mini-batches $\in R^{\left(Bsize\times T\times T\times IJ\right)}$.

(2) $$\begin{array}{c} GADF=\phi ⊗\psi -\psi ⊗\phi ,\;\;GADF\in R^{\left(T,T\right)}\\ \phi ={\left\{\sqrt{\frac{\phi_{interm.,i}-\min\phi_{interm.}}{\max\phi_{interm.}-\min\phi_{interm.}}}\right\}}_{i=1}^{n},\;\phi \in R^{\left(T\right)}\\ \phi_{interm.}={\left\{1-\psi_{i}^{2}\right\}}_{i=1}^{n},\;\;\psi \in R^{\left(T\right)},\;\;\phi_{interm.}\in R^{\left(T\right)} \end{array}$$

### 3.2. Feature Extractor

The goal of the feature extractor (i.e., backbone network) is to map the input modality to a latent representation consisting of features that are relevant for activity/gesture recognizer and similarity discriminator blocks. Our feature extractor is based on mobile inverted bottleneck [30] and squeeze-an-excitation blocks [31]. Block details are omitted but can be found in [30,31]. The listed hyperparameters were obtained via hyperband optimization [32], for which an epoch budget of 600 per iteration, 5 iterations, and a model discard proportion per iteration of 3 were used.

Most hyperparameters of the feature extractor used together with DFS as input type can be found in Table 1. The standard convolution layers denoted in Table 1 are initialized by random numbers drawn from a variance-scaled normal distribution and consider no in-between pooling and bias addition [33]. We apply padding to keep input and output sizes the same (e.g., ‘same’ padding). The variance-scaled normal distribution uses scale factor 2 and ‘fan_out’ mode. The max. pooling layer denotes a global max. pooling layer (pool size engulfs the entire input width/height, and max. pooling operation is only applied once). None of the MobileV2 blocks use dropout regularization or batch normalization. MobileV2 block weights are initialized randomly by drawing from a variance-scaled normal distribution with a scale factor of 2 and ‘fan_out’ mode.

Most hyperparameters of the feature extractor used together with GADF as input type can be found in Table 2. The start and end convolution operation weights are initialized by random numbers drawn from a variance-scaled normal distribution (layers do not consider bias addition). This distribution uses scale factor 2 and ‘fan_out’ mode. Both operations consider padding to keep in/output sizes the same (e.g., ‘same’ padding). All max. pool layers, except for the last layer, also consider padding to keep in/output sizes the same. The last max. pooling operation to create the latent representation is a global max. pooling operation. None of the MobileV2 blocks use dropout regularization or batch normalization. MobileV2 block weights are initialized randomly by drawing from a variance-scaled normal distribution with a scale factor of 2 and ‘fan_out’ mode.

### 3.3. Activity Recognizer

The activity recognizer predicts gestures, i.e., it returns discrete probability distribution ${\left\{P\left(y_{i}|x\right)\right\}}_{i=1}^{n_{gestures}}$. Our activity recognizer is a Multi-Layer Perceptron (MLP) with two dense layers. The first layer contains 400 neurons with DFS as the input type when considering either the Widar3 or SignFi dataset. The first layer contains 128 neurons with GADF as the input type when considering the Widar3 dataset [13]. When considering GADF as the input type and the SignFi dataset, the first layer contains several neurons equal to the number of gesture classes available in the respective benchmark dataset. In other words, when considering GADF as the input type, the first layer contains a different number of neurons when considering the Widar3 or SignFi dataset. It is unwise to consider 128 neurons with GADF as the input type and the SignFi dataset. In this case, layer chaining from the first to the second layer ’upscales’ the number of neurons between the aforementioned layers. Upscaling typically results in suboptimal classification performance. The need for ’upscaling’ is caused by unanticipated variance in intermediate representation feature vector size between considered benchmark datasets. This is considered to be a methodology limitation. The activity recognizer considers ReLU to be an activation function. The second layer contains an equal number of neurons to the number of gesture classes present in every dataset (see Section 4.1). It also uses SoftMax activation to create a discrete probability distribution. Weights and biases are initialized via random numbers drawn from a variance-scaled uniform distribution with a scale factor of 1/3 and ‘fan_out’ mode. The hyperparameters described above were obtained via manual hyperparameter tuning.

### 3.4. Similarity Discriminator

The similarity discriminator computes scalars representing the similarity between two feature vectors. In the case of mini-batch alignment, the feature vectors are sampled probability density values at a value interval between outer distribution quantiles. Therefore, the feature vectors denote discrete 1D PDF approximations (referred to as 1D PDFs in the text). Its output is ignored during the inference stage. The discriminator uses a PDF estimation layer and a bilinear similarity layer. The input to the PDF estimation layer, $Z\in R^{Bsize\times F}$, is a concatenation of the feature extractor output and the activity recognizer output *F*. Concatenation is preferred since the activity recognizer output contains domain-specific features but is still helpful to the activity-recognition task [11]. Per feature in *F*, the PDF estimation layer uses Kernel Density Estimation (KDE) to create a 1D probability distribution across values from different mini-batch samples (in Equation (3), the KDE procedure is illustrated in mathematical form). The PDF is sampled across a range of values, in which the outer values are the zeroth and fourth distribution quantiles. The equation assumes the existence of one value vector $z\in Z^{⊤}$. h>0 is a smoothing parameter that should be set such that important PDF peaks are not obscured, and random PDF peaks are filtered out. Equation (3) can be generalized to a multivariate situation by considering *m* to be a d-variate value coordinate vector, *h* to be a smoothing matrix, considering the entire value vector *z*, and making use of multivariate kernels.

(3) $$\begin{array}{c} PDF\left(m\right)=\frac{1}{hBsize}\sum_{j=1}^{Bsize}K\left(\frac{m-z_{j}}{h}\right),\;K\left(\frac{m-z_{j}}{h}\right)=\frac{1}{\sqrt{2\pi }}e^{\frac{-{\left(\frac{m-z_{j}}{h}\right)}^{2}}{2}},\\ min(z)\leq m\leq max(z) \end{array}$$

We consider the artificially permuted PDFs per feature in *F*, created with the help of a diffeomorphism, as $PDFs^{+}$ (the PDF permutation process in mathematical form per feature is illustrated in Equation (4)). A diffeomorphism is a smooth, differentiable, and invertible element-wise map function denoted by *g*. The PDF estimation layer randomly picks between the following diffeomorphism with equally divided probability: scaling by scalar 2, shifting by scalar −3, reciprocal, chain shifting by scalar 5, and scaling by scalar 0.8, sigmoid, softplus, or exponential function. Because in total, 7 diffeomorphisms are considered, every diffeomorphism has a 14.3% chance of being picked. Diffeomorphism-picking is performed by randomly sampling an integer number in a range of which the min. bound is 1, and the max. bound is equal to the total number of considered diffeomorphisms. Randomly picking among a large set of diffeomorphisms results in more PDF permutation variety and makes similarity loss maximization difficult. The difficulty can be found in less bias towards a severely limited number of diffeomorphisms. The first-order derivative of the inverse map function can be generalized to a multivariate situation by substituting the first-order derivative with the determinant of the Jacobian matrix (i.e., matrix of all first-order partial derivatives) [34]. We do not consider multivariate PDFs due to non-linear scaling issues this has caused during experimentation (see Section 5.5).

(4) $$\begin{array}{c} PDF^{+}\left(v\right)=PDF\left(g^{-1}\left(v\right)\right)+\left|{\left(g^{-1}\right)}^{{}^{\prime }}\left(v\right)\right|,\;min\left(g\left(z\right)\right)\leq v\leq max\left(g\left(z\right)\right) \end{array}$$

The PDF estimation layer contains a bank $\in R^{\left(2+C\times F\times R\right)}$ (i.e., persistent tensor across mini-batches, epochs) made of PDFs from previously encountered mini-batches, denoted by $PDFs^{-}$. In the first two matrix indices, the PDFs and permuted PDFs of the current considered mini-batch are stored. Prior to copying the PDFs into the first matrix index, the previous PDFs are assigned to a random other matrix index in the bank. The bank capacity *C* denotes the maximum number of other matrix indices. *R* is the PDF sampling resolution (i.e., the number of evenly spread PDF samples in the range between the zeroth and fourth distribution quantiles). Prior to training, the entire bank is randomly initialized. During training, the bank is fed to the bilinear similarity layer. The PDF estimation layer uses a bank capacity of 40, a smoothing (bandwidth) parameter of 0.335, and a PDF sampling resolution of 100. The bank and square matrices per feature in *F* in the similarity layer are initialized with random numbers drawn from a variance-scaled uniform distribution with a scale factor of 1/3 and ‘fan_out’ mode. The similarity discriminator hyperparameters were determined via manual hyperparameter tuning.

The bilinear similarity layer, per feature in *F*, learns a similarity function $sim\left(a,b\right)=a^{⊤}Wb$, where $W\in R^{\left(R\times R\right)}$ is a square matrix. Its output ($\in R^{\left(1+C\times F\right)}$) for every feature in *F* contains one similarity scalar $sim(PDF,PDF^{+})$ and *C* similarity scalars $sim(PDF,PDF^{-})$.

### 3.5. Overall Adversarial Loss

We train the mini-batch alignment network via adversarial learning. A task loss associated with the activity recognizer is minimized, and a similarity loss associated with the similarity discriminator is maximized. The overall loss is given by Equation (5). $|\hat{Y}|$ denotes the batch size, $c_{a}$ the gesture classes, $y_{i,j}$ the true gesture class label value (0 or 1), and ${\hat{y}}_{i,j}$ the predicted gesture class label value (0–1). β is a weight (a hyperparameter) that controls the impact of the similarity loss. Without this weight, the similarity loss takes over cost minimization, therefore causing the overall loss to diverge. Symbol λ denotes a weight that controls the impact of matrix element values on the overall loss. It should be > 0 to have an effect and not too large to avoid classification loss overfitting and overall loss explosion.

(5) $$\begin{array}{cc} L=\; & -\frac{1}{|\hat{Y}|}\sum_{i=1}^{|\hat{Y}|}\sum_{j\in c_{a}}y_{i,j}\ln{\hat{y}}_{i,j}\;+\;\frac{\beta }{F}\sum_{F}\ln\frac{e^{sim(pdf,pdf^{+})}}{\sum_{C}e^{sim(pdf,pdf^{-})}}\; \end{array}$$

## 4. Experimental Setup and Performance-Evaluation Strategy

In addition to the input types described in Section 3, the performance-evaluation experiments for mini-batch alignment gesture classification also include the utilization of two gesture-recognition datasets and four neural network pipelines. These experiments aim to compare the performance of gesture classification among these different pipelines. We are interested in general performance across different dataset subset splits when there is a clear domain shift between data used for training and data used during gesture-recognition testing. We artificially introduce domain shift between the hold-out data subset for gesture-recognition testing and the other subset used for training by employing a performance cross-validation (CV) technique called leave-one-domain-factor-out CV. This means that if data were collected with the help of 5 test subjects, for example, data belonging to one test subject would be placed in the data subset used for gesture-recognition testing, and data belonging to the other test subjects would be placed in the data subset for training. A specific data subset split refers to which test subject data are placed into the data subset used for gesture-recognition testing. The domain-shift introduction is considered to be artificial because in real-life data-inference scenarios, the change from a specific set of domain factors to another, causing domain shift, happens non-deterministically. We consider artificial domain-shift introduction to be a limitation of experimentation.

The comparison between the gesture classification performance of mini-batch alignment and the results reported in previous studies [12,13] is conducted by examining the trends in the results. For example, a trend may indicate that including attention in the neural network pipeline yields better performance compared to not considering a technique for mitigating domain shifts under leave-one-user-out cross-validation. By focusing on trends rather than directly comparing quantitative gesture classification performance results, this approach ensures that valid and fair comparisons are made, preventing comparing disparate or incomparable results.

### 4.1. Gesture-Recognition Datasets

For our experiments, we used the SignFi [15] and Widar3 [13] datasets. The SignFi dataset consists of Wi-Fi–CSI data captured while subjects under test perform many everyday sign gestures. The domain factors present in the dataset include two different environments, i.e., a home and a lab environment, and five different human test subjects. The lab environment has a much greater surface area (156 m${}^{2}$ versus 15.6 m${}^{2}$) in comparison to the home environment and contains many more desks and cabinets. It also does not contain items such as a bed or closet in comparison to the home environment. The test subjects vary in age, height, and weight, in ranges of 26–39 years, 168–180 cm, and 55–90 kg, respectively. Wi-Fi–CSI frames inside the dataset are assumed to be already pre-processed tensors $\in C^{\left(3,30,200\right)}$. Random phase shifts caused by sampling clock and carrier frequency offsets between Wi-Fi senders and receivers were removed by the SignFi dataset creators by adding a term to the raw Wi-Fi–CSI phase tensor constructed via a multiple linear regression task. The SignFi dataset creators then unwrapped the phase values inside the Wi-Fi–CSI phase tensor. The pre-processed Wi-Fi–CSI phase tensor and raw Wi-Fi–CSI amplitude tensor were then combined by the SignFi dataset creators into a complex pre-processed Wi-Fi–CSI tensor [15].

Our experiments with the SignFi dataset involve both user/environment domain factor leave-out cross-validation. During environment leave-out cross-validation, the downlink home and lab data subsets for user 5 were considered. The home data subset contains 2760 samples (276 sign gestures · 10 repetitions), and the lab data subset has 5520 samples (276 sign gestures · 20 repetitions). During user leave-out cross-validation, a downlink lab data subset for users 1–5 was considered. This data subset contains 7500 samples (150 sign gestures · 5 users · 10 repetitions).

From the original Widar3 dataset, we made a subset including all repetitions of the following 6/22 gestures: push&pull, sweep, clap, slide, draw-O (horizontal), and draw-zigzag (horizontal). Regarding domain factors, we used data from environment 1 (classroom), all torso locations and face orientations, and user ids 10, 12, 13, 14, 15, and 16. Users vary in their age, weight, and height, in ranges of 22–28 years, 45–87 kg, and 155–186 cm, respectively. The total size of this subset dataset is 6gestures·5gesturerepetitions·5torsolocations·5faceorientations·6userids=4500samples. For every domain factor leave-out cross-validation experiment, the same Widar3 data subset was used. The reason for considering the data subset settings described above is that several research projects such as [12,35] construct a Widar3 subset dataset using similar data subset settings for their experiments. The use of the same settings closely allows a fair comparison between our results and theirs.

### 4.2. Domain-Shift Mitigation Techniques

**Figure 2:**
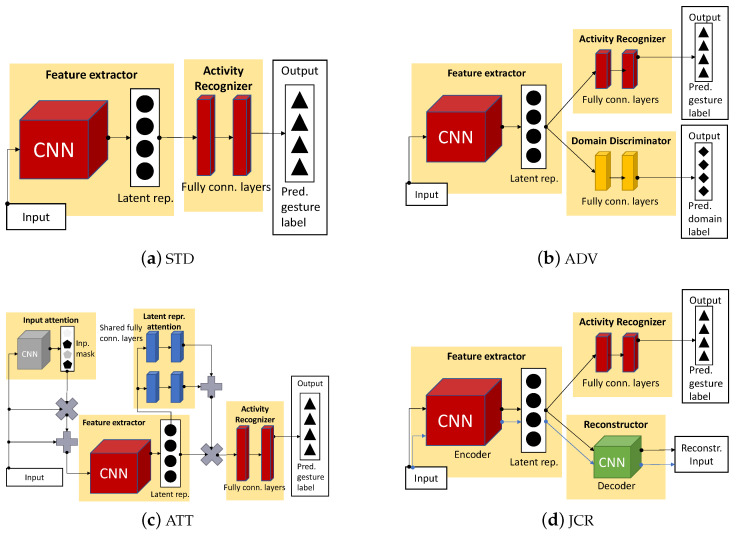
Neural network pipeline summaries of pipelines to which the gesture classification performance of mini-batch alignment is compared. (**a**) standard classification (STD), (**b**) adversarial domain classification (ADV), (**c**) input and intermediate representation attention (ATT), and (**d**) joint classification and reconstruction (JCR). Further details, including hyperparameters, can be found in Section 4.2.

We compare our mini-batch alignment technique against four other neural network pipelines. These pipelines are: (i) standard classification (i.e., no domain-shift mitigation) denoted as STD, (ii) adversarial domain classification denoted as ADV, (iii) joint classification and reconstruction denoted as JCR, and (iv) input and intermediate representation attention denoted as ATT. The pipelines considered in our performance-evaluation experiments for benchmark comparison can be found in Figure 2. The feature extractor and activity recognizer hyperparameters of the neural network pipelines in Figure 2 are similar to the hyperparameters listed in Section 3 and are therefore not addressed in this subsection. The hyperparameters that are addressed in this section are similar for both the DFS and GADF input types unless stated otherwise.

#### 4.2.1. No Domain-Shift Mitigation (STD)

No domain-shift mitigation (i.e., standard classification) considers a neural network that only uses the pre-processing, feature extractor, and activity recognizer blocks presented in Section 3. It only predicts gestures, i.e., discrete probability distribution ${\left\{P\left(y_{i}|x\right)\right\}}_{i=1}^{n_{gestures}}$. It is trained via standard loss minimization (the loss in mathematical form is illustrated in Equation (6)).

(6) $$\begin{array}{c} L=-\frac{1}{|\hat{Y}|}\sum_{i=1}^{|\hat{Y}|}\sum_{j\in c_{a}}y_{i,j}\ln{\hat{y}}_{i,j} \end{array}$$

#### 4.2.2. Adversarial Domain Classification (ADV)

The adversarial domain classification neural network pipeline substitutes the similarity discriminator presented in Section 3 by a domain discriminator. The domain discriminator predicts a set of domains, i.e., it returns discrete probability distribution ${\left\{P\left(s_{j}|x\right)\right\}}_{j=1}^{n_{domains}}$. During testing, the domain discriminator output is ignored.

The domain discriminator is an MLP of four dense layers. The reason for having four layers is that having two layers (the same number as the activity recognizer) did not provide enough domain discriminator sub-network complexity for the domain classification network, therefore not showing performance metric results rising above the standard classification network. Sub-network complexity is a term used to describe the degree of intermediate feature space non-linearity to which the domain discriminator can fit a function according to a desired loss outcome without under-/overfitting. The term description assumes that under-/overfitting is not caused by insufficient training time or dataset volume. The layers contain 400, 300, and 200, and the number of unique domain factor combination neurons when DFS was used as the input type. When GADF was used as the input type, the number of neurons was 160, 150, and 150, and the number of unique domain factor combinations, respectively. The first 3 layers use ReLU activation, and the last layer uses SoftMax activation. Weights and biases are initialized via random numbers drawn from a variance-scaled uniform distribution with a scale factor of 1/3 and ‘fan_out’ mode. The domain discriminator hyperparameters listed above were determined via manual hyperparameter tuning. Adversarial training was employed, in which a task loss associated with the activity recognizer was minimized, and domain loss associated with the domain discriminator was maximized as shown in Equation (7). Symbol $|\hat{S}|$ denotes the batch size, $c_{d}$ the domain classes, $s_{i,j}$ the true domain class label value (0 or 1), and ${\hat{s}}_{i,j}$ the predicted domain class label value (0–1).

(7) $$\begin{array}{c} L=-\frac{1}{|\hat{Y}|}\sum_{i=1}^{|\hat{Y}|}\sum_{j\in c_{a}}y_{i,j}\ln{\hat{y}}_{i,j}+\frac{\beta }{|\hat{S}|}\sum_{i=1}^{|\hat{S}|}\sum_{j\in c_{d}}s_{i,j}\ln{\hat{s}}_{i,j} \end{array}$$

#### 4.2.3. Joint Classification and Reconstruction (JCR)

The joint classification and reconstruction pipeline substitutes the similarity discriminator presented in Section 3 with a reconstructor block having a decoder CNN and two input flows, i.e., a source (black arrows) and target (blue arrows) flow. Flows are not the same as input modalities, as each uses the same single input modality propagated sequentially. In the case of using DFS as the input type, the current input DFS sample is given as the source flow, and a randomly picked input DFS sample is given as the target flow to the model. Chen et al. [9] explained that unlabeled input samples coming from a much larger target dataset involving many more domain factors are normally considered for the target flow. However, as our dataset is small, this target data requirement cannot be met.

In a single training run using a source and target sample, first, a classification and reconstruction loss was computed based on the source sample simultaneously. The classification loss was computed with a categorical distribution (pipeline prediction) and a one-hot encoded class label. The reconstruction loss was computed with the original source sample and a version reconstructed by the decoder CNN. Subsequently, a reconstruction loss was computed with the target sample and a reconstructed version thereof. The sum of reconstruction losses for both flows gives the overall reconstruction loss. Partial loss derivatives were computed for both classification and overall reconstruction loss and used to update trainable model parameters of the model inside the pipeline separately in a sequence (see [9] for more details). The classification loss is similar to the loss expressed in Equation (6). The overall reconstruction loss $L_{r}$ is expressed in Equation (8).

(8) $$\begin{array}{c} L_{r}=\frac{1}{|\hat{X}{|}_{s}}\sum_{i=1}^{|\hat{X}{|}_{s}}{\left(x_{s,i}-{\hat{x}}_{s,i}\right)}^{2}+\frac{1}{|\hat{X}{|}_{t}}\sum_{i=1}^{|\hat{X}{|}_{t}}{\left(x_{t,i}-{\hat{x}}_{t,i}\right)}^{2} \end{array}$$

The decoder CNN inside the reconstruction block is connected to the output of the feature extractor. Most of the decoder is an exact reverse copy of the start and the end 2D convolution layers and mobile inverted bottleneck and squeeze-and-excitation blocks in the feature extractor. Prior to being given to the decoder CNN, the intermediate representation is first upsampled to the tensor shape prior to being created via global max. pooling by means of nearest interpolation.

Most decoder hyperparameters used in the case of using DFS as input type can be found in Table 3. Inside the decoder, an altered version of the mobile inverted bottleneck and squeeze-and-excitation block is used. The input to the 2D depth-wise convolution layer is upsampled by a factor 2 × 2 via the nearest interpolation. The 2D depth-wise convolution layer stride is set to 1 × 1. All convolution layers/blocks in the decoder, except the squeeze-and-excitation layers inside the MobileV2 block, consider no bias addition. Padding is applied to all decoder layers where appropriate to keep input and output sizes the same. The reason final reverse convolution layer construction deviates from the upsampling and normal convolution paradigm is that upsampling with a sample size 2 × 8 in combination with a channel output much larger than the original input channel size creates a tensor so large it cannot be stored in GPU memory. All decoder weights were initialized by random numbers drawn from a variance-scaled normal distribution. The variance-scaled normal distribution uses scale factor 2 and ‘fan_out’ mode.

Most hyperparameters used in the case of using GADF as input type can be found in Table 4. Inside this decoder, an altered version of the mobile inverted bottleneck and squeeze-and-excitation block is used. The input to the 2D depth-wise convolution layer is not upsampled (an upsampling layer with a factor 1 × 1 via nearest interpolation is considered). The 2D depth-wise convolution layer stride is set to 1 × 1. None of the decoder layers, except for the convolution layers in the squeeze-and-excitation portion of the MobileV2 block, consider bias addition. Every decoder layer, when applicable, uses padding to keep input and output sizes the same. All decoder weights are initialized by random numbers drawn from a variance-scaled normal distribution. The variance-scaled normal distribution uses scale factor 2 and ‘fan_out’ mode.

#### 4.2.4. Input and Intermediate Representation Attention (ATT)

The input and intermediate representation attention pipeline has a neural network comparable to the neural network of the standard classification pipeline except for the addition of two attention blocks, i.e., input and intermediate representation attention. The purpose of the input attention block is to create a single input sample representation, which, when combined with the original input sample, mitigates irrelevant input sample parts, therefore optimizing the overall loss minimization process further. The purpose of the intermediate representation attention block is to find important correlations between intermediate representation features across time and put more emphasis on these correlations in subsequent neural network components. The attention blocks are simultaneously trained with the other neural network components end-to-end, which involves a standard classification loss, which can be found in Equation (6). More elaboration on the attention blocks can be found in [12].

Most input attention block hyperparameters can be found in Table 5. Batch normalization is applied on the block input first, prior to the convolution layer. The convolution layer does not consider bias addition, applies padding to keep input and output sizes the same, and its weights are initialized by random numbers drawn from a variance-scaled normal distribution. The variance-scaled normal distribution uses scale factor 2 and ‘fan_out’ mode. The output of the convolution layer is subsequently element-wise multiplied with the block input. The output of the multiplication operation is subsequently used in an element-wise addition operation with the block input. The output of the addition operation is finally passed on to the feature extractor (see Figure 2).

The intermediate representation attention block starts by forking the intermediate representation into two (see Figure 2). Subsequent operations, including hyperparameters, for each fork can be found in Table 6, where forks are denoted by F1 and F2. Denoted pooling operations consider padding to keep input and output sizes the same. After the pooling operations, the pooling operation outputs are passed through an MLP. This MLP is denoted as shared MLP inside the table. The fully connected layer weights and biases are shared between the MLPs for each fork. Fully connected layer weights are initialized by random numbers drawn from a variance-scaled normal distribution. The variance-scaled normal distribution uses scale factor 2 and ‘fan_out’ mode. The MLP outputs are subsequently combined (forks are merged) via an element-wise addition operation. The output of the addition operation is passed through a sigmoid activation function. The output of the activation function is subsequently element-wise multiplied with the intermediate representation coming out of the feature extractor. The output of the multiplication operation is given to the activity recognizer MLP (see Section 3.3).

### 4.3. Performance-Evaluation Strategy

For neural network model training, we used Adaptive Moment Estimation (ADAM) optimization [36] with a learning rate of 0.0001, number of epochs 100, and batch size 12, respectively, for all experiment combinations (domain-shift mitigation technique, input type, etc.). β was set to $1\cdot 10^{-8}$ for mini-batch alignment and $1\cdot 10^{-6}$ for adversarial domain classification. These hyperparameters were determined via manual hyperparameter tuning. For reproducibility purposes, for all operations involving a random component, pseudo-randomness was introduced using a random seed of 42. During training, early stopping was used, where every training procedure considered overall validation subset loss as a stopping criterion. The minimum delta was set to 0 and patience to 5 due to encountering a rippling validation loss. In addition, when subsequent validation loss results do not show an improvement, neural network trainable parameter updates are not saved (i.e., best-performing trainable parameters are always kept).

Prior to training, datasets were split into training, validation, and test subsets. As explained before, the performance-evaluation experiments use leave-one-domain-factor-out cross-validation. For example, when considering the environment as a factor to be left out and SignFi as the dataset, the home data subset for user 5 was considered for training the lab data subset for user 5 for testing, and vice versa. When considering the user as the factor to be left out and Widar3 as the dataset, $\tilde{1}6.7$% of the dataset belonging to a respective user was placed in the test subset, and the data belonging to the other users was placed in the training subset. When considering the SignFi dataset, the training subset was further split randomly into a 90% training subset and a 10% validation subset via stratified sampling based on the task label. When considering the Widar3 dataset, 5 torso locations · 5 face orientations · 6 user ids = 150 unique domain combinations exist. When leaving out the user, 125 of these combinations exist inside the training subset. From these combinations, 90% was selected randomly and used to place samples inside a re-sampled training subset based on domain label. The other 10% was used to sample a validation subset based on domain labels. The reason domain labels were not considered for sampling the training subset into a training and validation subset together with the SignFi dataset is due to the limited number of environment and user domain factors present in the SignFi environment factor data subset (in the training data subset you have 1 environment and 1 user which leads to a single unique combination which cannot be used for data splitting).

After training, the gesture classification performance was evaluated via inference with a set of performance metrics and the test subset. Performance metrics considered include accuracy (A), precision (P), recall (R), F1 score (F), and Kappa score (CK).

## 5. Results and Discussion

In what follows, performance-evaluation and cross-comparison results of the mini-batch alignment technique pipeline, all the aforementioned benchmark pipelines, and the results reported in previous studies are presented and discussed. These results, as well as pitfalls contributing to bad MBA results, are further explained.

### 5.1. One-Domain-Factor Leave-Out Cross-Validation

**Figure 3:**
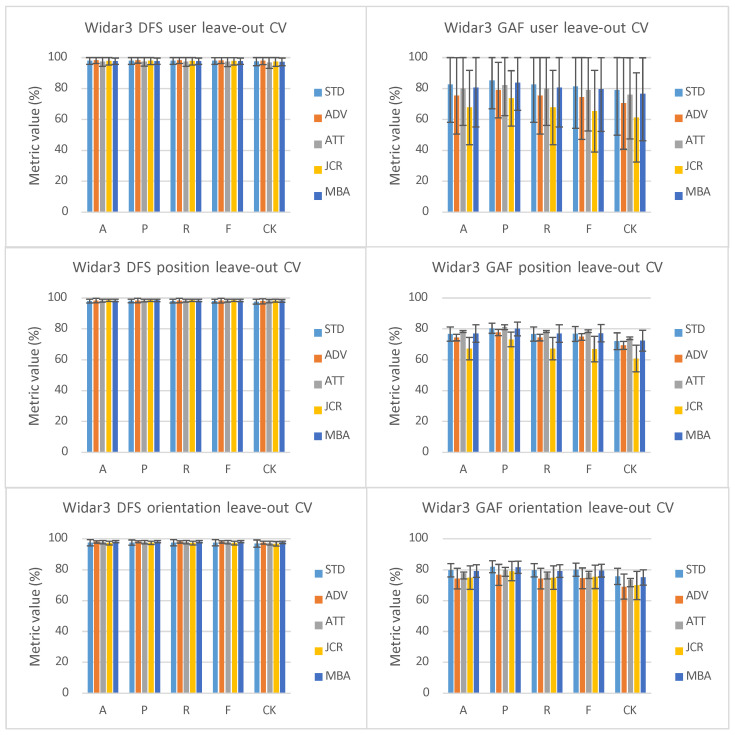
Widar3 subset—sampled test subset performance metric result comparison based on one-domain-factor leave-out cross-validation. GAF denotes the 4× reduced GADF input type as explained in Section 3.1.

Looking at the user leave-out results in Figure 3 and considering DFS as input type, a smaller, therefore better, variance range can be seen for ADV across all performance metrics. Additionally, the mean P and CK values slightly outperform STD. All other domain-shift techniques either have a larger, therefore worse, variance range and/or mean performance metric values that are on par or do not outperform STD. The position leave-out results present a different story, with all domain-shift techniques’ mean performance metric values being slightly better than STD. However, ADV has a larger variance range in comparison to the other domain-shift techniques across all performance metrics. The results of other domain-shift techniques are approximately similar to each other. Orientation leave-out results also indicate that all domain-shift techniques’ mean performance metric values are slightly better than STD. JCR and ATT perform slightly worse in comparison to ADV and MBA. Interesting to note is that for all Widar3 DFS results, STD already has very good performance results. Therefore, the introduction of the domain-shift mitigation technique will not lead to drastic performance improvements in this scenario. This is likely caused by the squeeze-and-excitation system in the EfficientNet blocks inside the feature extractor (see Section 3.2). This system functions as a type of channel-based attention and, therefore, already reduces domain shift for a large portion. A comparison summary can be found in Table 7.

Looking at the user leave-out results in Figure 3 and considering 4× reduced GADF as input type, on average, all domain-shift mitigation techniques perform worse compared to STD. This indicates that domain-shift mitigation techniques, when combined with simple memory complexity reduction techniques such as interpolation, can lead to nondeterministic reductions in inference performance in artificially introduced domain-shift scenarios. We consider potential performance improvement gains resulting from (1) considering DFS as input type and (2) removing input interpolation suboptimal. This is due to the vast increase in required memory complexity and subsequent computational complexity due to having to convolve over a much larger tensor structure, therefore needing much higher compute and memory service requirements during network training. An exception to this is the slightly better ATT results and on-par MBA results in comparison to STD for leave-one-position-out cross-validation. This indicates that ATT’s focus blocks were able to recuperate some of the lost performance by filtering out irrelevant input features/intermediate representation feature correlations. The MBA result may either mean (i) MBA does not mitigate any domain shift at all, therefore achieving a result on par with STD, or (ii) it is able to recuperate performance to the extent that no additional performance due to weight steering is lost. The overall large variance range observed for one-user-factor leave-out cross-validation is caused by a single user being left out, for which a much lower performance metric result is observed in comparison to all other users being left out. We consider the most likely explanation for this to be that the linear interpolation reduces those input features that are important for placing samples belonging to this user inside the correct class distribution in higher-level feature representation layer output spaces. Domain changes across different data-collection moments are an unlikely culprit since domain-shift mitigation techniques under test would have absolved those issues; however, it cannot be ruled out completely based on the performance results alone.

**Figure 4:**
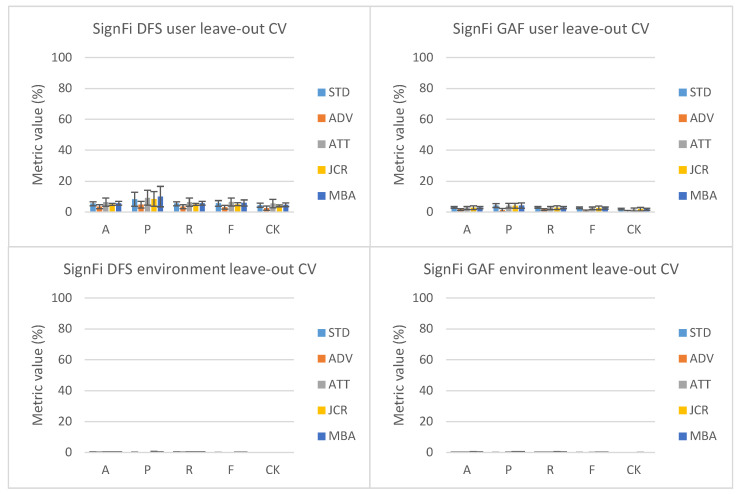
SignFi dataset-sampled test subset performance metric results based on one-domain-factor leave-out cross-validation. GAF denotes the 4× reduced GADF input type as explained in Section 3.1.

Looking at the SignFi leave-one-domain-factor-out cross-validation results in Figure 4, it can be noted that for none of the domain-shift mitigation techniques, including STD, any noteworthy performance above the random guessing threshold of approximately 30% was achieved. These results indicate either (i) severe discriminative differences between training/validation and test set samples belonging to a similar task class or (ii) a badly configured input pre-processing pipeline causing input sample value invariances leading to fitting problems. To determine which of the two led to the performance metric values, we had a closer look at the training and validation loss progression for one of the user-factor leave-out cross-validation splits when considering JCR as domain-shift mitigation technique (see Section 5.3).

### 5.2. Two-Domain-Factor Leave-Out Cross-Validation

One-domain-factor leave-out results with DFS as input type revealed that STD already has very good performance results (see Section 5.1). For the two-domain-factor leave-out experiments, we did not continue with the SignFi dataset due to its poor performance (not exceeding the random guessing). Only orientation is considered to be a domain factor type to be left out to speed up subsequent experimentation. This means that two-domain-factor leave-out does not refer to leaving two domain factors belonging to different domain factor types (e.g., one-orientation- and one-user-factor-out).

The one-domain-factor leave-out sampling scenario for the Widar3 dataset was explained in Section 4.3. Changes made when considering two-domain-factor leave-out CV are subsequently explained. Rather than placing data from a single respective user in the test subset (16.7%), data from two respective orientation factors is placed in the test subset (40%). The other data (60%) is placed in the training subset. This training subset is used in the subsequent training and validation subset sampling procedure. With 5 orientation domain factors, in total, 5·5=25 domain factor combinations can be made. From these 25, 5 are picked pseudo-randomly. This leaves 90 unique domain combinations to be used for further sampling the training data subset into a training and validation data subset. More information regarding this sampling procedure can be found in Section 4.3 since the parts of the sampling procedure not touched upon in this subsection remain the same.

**Figure 5:**
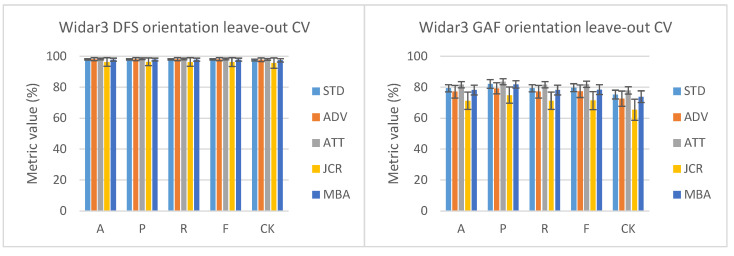
Widar3 subset—sampled test subset performance metric result comparison based on two-orientation-factor leave-out cross-validation. GAF denotes the 4× reduced GADF input type (see Section 3.1).

Looking at the Widar3 DFS leave-two-orientation-factors-out cross-validation results in Figure 5, ATT performs better across both variance range and mean performance metric value w.r.t all other domain-shift mitigation techniques. This means that ATT is better able to deal with larger domain shifts under the presumption that this also causes the amount of available training data to be lower. JCR shows a large performance drop regarding all other domain-shift mitigation techniques. This indicates that if the amount of unseen domain shift increases and the amount of available training data decreases, domain-adaptation techniques such as JCR should ideally be avoided. Both ADV and MBA perform slightly worse regarding STD when looking at the performance metric value variance range. The observation that the introduction of the domain-shift mitigation technique will not lead to drastic performance improvements also holds for a leave-two-orientation-factor leave-out cross-validation scenario. A comparison summary can be found in Table 8.

Looking at the two-orientation leave-out results in Figure 5 with 4× reduced GADF as input type, on average, all domain-shift mitigation techniques also perform worse in comparison to STD. This means that simple memory complexity reduction techniques such as interpolation can also lead to nondeterministic reductions in inference performance in a two-orientation leave-out cross-validation setting. In this case, MBA is not able to keep performance metric value results on par with STD. This hints at potentially extra performance reductions caused by MBA steering the feature space. ATT still slightly outperforms STD on both variance range and mean performance metric value. Therefore, the observation that ATT’s focus blocks were able to recuperate some of the lost performance also holds in a two-orientation-factor leave-out cross-validation scenario.

### 5.3. SignFi Fitting Problems

**Figure 6:**
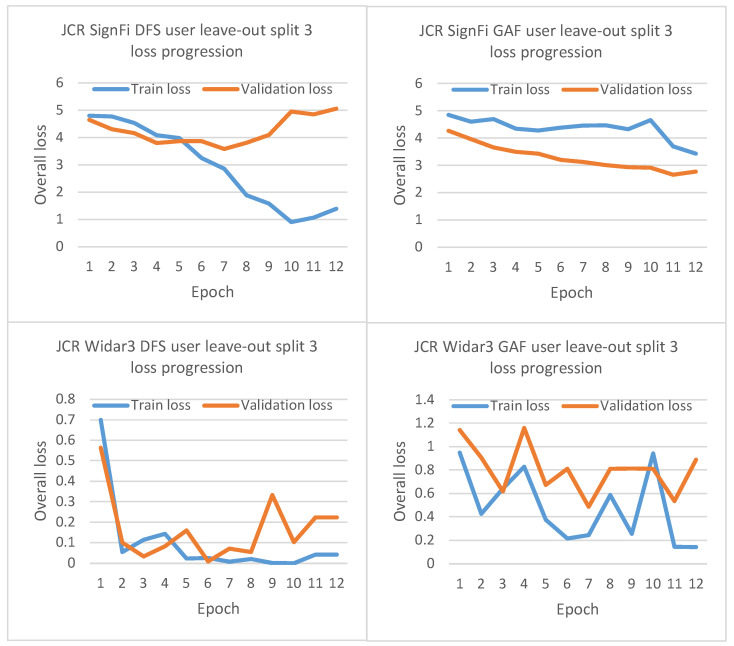
Widar3 subset and SignFi dataset-sampled train and validation subset loss progression result comparison based on one-user-factor leave-out cross-validation split 3 from range 0–4 and JCR as domain-shift mitigation technique. GAF denotes the 4× reduced GADF input type as explained in Section 3.1.

As mentioned in Section 5.1, SignFi results show no noteworthy performance improvement above a typical random guessing threshold. In Figure 6, the overall loss progression history for JCR during leave-one-user-out cross-validation split 3 (in the range between 0 and 4 for the SignFi dataset and 0–5 for the Widar3 dataset) is shown. Looking at the Widar3 loss progression history, it can be noted that for both DFS and GADF as input types, the loss calculated over both training and validation subset batches closely follows each other toward a minimum loss value. Therefore, the optimization algorithm correctly achieves its intended goal. The limited discrepancy between the two losses is caused by the inevitable required model trade-off between non-linear function variance toward new samples and bias towards observed samples.

However, looking at the SignFi loss progression history, it can be noted that for DFS as the input type, there is an extremely high bias toward the training dataset (overfitting). For GADF as input type, the model is not able to fit an appropriate function to both the train and validation subsets (underfitting). Both situations are unlikely to be caused by extreme discrimination towards training domains (factors) during training. Discrimination typically shows up as a large loss increase measured on a left-out test subset, while observed loss history on an in-domain sampled train and validation subset across time does show a minimization trend as observed with the Widar3 dataset.

One explanation is that the SignFi dataset contains a low number of samples per domain factor and class. The Widar3 dataset contains 750 samples per task class, while the SignFi user and environment factor datasets contain 40 and 20/10 samples per class, depending on which environment is left out. In such a situation, the chance that a class distribution in the intermediate representation encompasses either (i) a too-small space, therefore not being able to properly represent the entire space where correct training and validation samples belonging to the class may reside, or (ii) a space too specific to the training dataset therefore not properly representing unseen validation samples belonging to the class is much higher. In such a situation, n-shot learning involving a distance comparison between a set of n-query samples of a certain class and a sample under test is more appropriate.

A second explanation is a misalignment between the considered data-collection setup and considered pre-processing methods inside the model pipeline. When there is one receiver antenna, and the subject under test is standing very close to this antenna, observed multi-path components (higher reliance on the static environment for reflection/diffraction/etc.) and or receiver signal amplitude values (potential NLOS situation with values following unfavorable Rayleigh fading distribution) may become much more similar between samples, therefore, increasing the chance that a dependence on features is created which do not properly distinguish between classes. In this case, the use of phase values instead of amplitudes inside the pre-processing pipeline may be better.

### 5.4. Cross-Comparison One-Orientation Leave-Out Cross-Validation Deviation

Both [12,13] report a specific one-orientation leave-out cross-validation result deviation in comparison to other types of one-domain-factor leave-out cross-validation, such as position or user. Widar3 reports an average accuracy percentage deviation of approximately 7% [13] and WiGRUNT 3% [12]. Looking at the results in Figure 3, it can be noted that there is approximately 1% orientation leave-out cross-validation result deviation in comparison to other types of domain factor leave-out cross-validation for ADV and MBA when considering DFS as input type. The observations presented above suggest the following: a lower orientation leave-out cross-validation result deviation in comparison to other types of domain factor leave-out cross-validation can be achieved by incorporating newer neural network components (e.g., from standard CNN and LSTM components in the Widar3 network [13], to ResNet components in the WiGRUNT network [12], and to mobile inverted bottleneck and squeeze-and-excitation components in the mini-batch alignment network) in the backbone network block while involving DFS as input type.

### 5.5. Mini-Batch Alignment Pitfalls

Observations made in Section 5.2 hint at MBA potentially steering the feature space during model training. However, the obtained results do not provide enough evidence to support that MBA is able to solve domain-independent learning and/or domain adaptation. In what follows, we explain potential pitfalls that may have led to this.

#### 5.5.1. Issues with Benchmark Datasets

Publicly available Wi-Fi–CSI benchmark datasets, e.g., [13,15,37], lack sufficient domain factor information. Most of them do not contain information about which user, environment, and/or height factor was used when measuring the distance factor data and vice versa, for example. This lack of information also complicates domain factor leave-out cross-validation because correctly leaving out specific factors is impossible. Another problem is that most of the publicly available data is already pre-processed to a certain extent. Therefore, it is difficult to accurately apply pre-processing techniques that rely on knowing the original sampling frequency of the data. Lastly, publicly available benchmark datasets are currently limited to a small set of domain factors artificially introduced in experiment environments and outdated measurement platforms (e.g., Linux 802.11n, Atheros, and Nexmon CSI tools). Certain neural networks (see Section 6) use a domain label vector in which every vector element is a unique combination of domain factors. This implies that there is a certain interplay between domain factors in a specific environment that requires further exploration for collecting new datasets.

#### 5.5.2. Lack of Precise Methodology Descriptions

In several research papers, the methodology required for the correct implementation of the baseline pipelines and comparison between the aforementioned pipelines and the mini-batch pipeline is not clear. Most research papers only consider accuracy as a metric, which cannot discriminate between pipelines when data or feature sets are heavily skewed in class distribution [38]. Therefore, in addition to accuracy, performance metrics such as precision, recall, F1 score, and Kappa score should be included for a fair comparison with the mini-batch alignment pipeline.

#### 5.5.3. Invalid Probability Distribution Assumptions

Our results may suffer from feature selection bias since 1D PDFs per feature may not properly capture information to forget. For example, a copula [39] could be introduced as an additional feature for modeling the dependence structure between the 1D PDFs. Another issue may be not knowing the intermediate representation feature meaning since the mini-batch alignment network, like many counterparts, is a black box. This causes picking specific diffeomorphisms for mini-batch alignment to have a nondeterministic effect on inference performance in-domain factor leave-out cross-validation. Lastly, we assumed no need for manual PDF sampling operation reparameterization for valid backpropagation. This assumption should be validated theoretically by analyzing the backpropagation process of the mini-batch alignment pipeline. In mini-batch alignment, there is a gradient dependency between the stochastic sampling operation on the KDE-based distributions and multiply-accumulate operations that involve the learnable feature extractor parameters. The aforementioned multiply-accumulate operations are used to generate *Z*, the input to the PDF estimation layer. In backpropagation, when performing gradient computation, the stochastic sampling operation should be reparametrizable automatically to a stable operation. If the operation is not re-parametrizable and this is not handled manually via a reparametrization trick, the aforementioned stochasticity makes the gradient minimization process inside the similarity discriminator pipeline pathway unstable. A canonical example when considering Variational AutoEncoders (VAEs) is applying a reparameterization trick to a posterior distribution to stabilize a partial derivative of the ELBO loss [40].

#### 5.5.4. Non-Linear Scaling Issues

We explained in Section 3 that the mini-batch alignment pipeline constructs 1D probability distributions across values from different mini-batch samples per feature. Prior to this probability implementation, we tried incorporating features into a multivariate probability distribution. We noted that using a multivariate distribution quickly becomes problematic. When measuring a PDF over a multivariate distribution, there is exponential memory growth with the feature number per batch sample. Even lower dimensionality PDF mapping with an MLP was infeasible. Due to scaling steepness, the lower dimensionality had to become so small and the MLP depth so shallow that learning a reliable reduced representation was intractable. The scaling steepness is also required because the square matrix in the bilinear similarity layer scales quadratically with the feature vector size. We tried saving multivariate PDFs into a sparse matrix and approximating the square matrix in the bilinear similarity layer to a variable size convex union of 4-sparse matrix bases [41]. We abandoned the sparse matrix and square matrix approximation solution as multivariate PDF sparseness cannot be guaranteed, which causes learning instability.

## 6. Related work on Mitigation of Domain Factor Effect in Wi-Fi–CSI-Based Activity/Gesture Recognition

Early Wi-Fi–CSI-based activity-recognition research mainly relied on extensive pre-processing pipelines and conventional machine-learning algorithms without taking the effects of domain factors into account. Li et al. [17] employed multiple pre-processing steps, namely outlier removal, denoising via low-pass filtering, and taking the weighted moving average of the CSI signal to reduce in-band noises efficiently. A discrete wavelet transformation was then applied to the signal to extract features, after which they were fed into a K-Nearest Neighbors (kNN) classifier. Feng et al. proposed a Multiple Activity Identification System (MAIS) [42], which focuses on the classification of multiple activities from different users in the same environment using the kNN algorithm. The authors of [16] first applied subcarrier selection on the CSI data by selecting the subcarrier that had the largest average amplitude between antennas. Afterwards, the CSI signal was passed through a median filter and low-pass filter for noise removal. Various statistics (e.g., mean and standard deviation) from the signal were computed as features, which were subsequently used in combination with a set of SVMs to make a classification of sign gestures based on a weighted voting scheme.

The CNN model proposed by Virmani and Shahzad in WiAG requires training samples of all gestures in only one configuration (i.e., the same position and the same orientation) before being able to generate samples of all the gestures provided in all their possible configurations [43]. Then, using the generated virtual samples, WiAG performs a kNN-based classification for each configuration. The authors of SignFi [8] employed a 9-layer Convolutional Neural Network (CNN) with Stochastic Gradient Descent with Momentum (SGDM) as an optimization algorithm for gesture recognition. They justify the use of CNNs based on their ability to automatically infer features with convolution operations that consider spatially local feature dependencies for computing output features in all spectrogram matrix index directions. Wang et al. [44] manually annotated each activity and labeled them, after which they upsampled the segmented CSI fingerprints to make them the same size in the time dimension using linear interpolation. Subsequently, CSI fingerprint matrices, stacked in the subcarrier dimension, are fed into a ResNet CNN model considering one-dimensional convolutions across the time dimension. Features computed only across the time dimension were highly correlated with activities to be classified. The authors of [44] introduced a joint activity recognition and indoor localization deep-learning framework and proposed a dual-task 1-D CNN with a re-implemented ResNet architecture created specifically for CSI fingerprints. Memmesheimer et al. [45] utilized EfficientNet to classify images such as RGB streams and skeleton estimates of individuals performing actions resulting from the encoding of multivariate signal sequences.

### 6.1. Domain Adaptation

Adaptation techniques aim to take the parameters of a neural network model (trained with data that a specific set of domains has altered) and tailor them to an unseen domain(s). Yang et al. [22] used a Siamese network consisting of two-time separated CNN + Bidirectional Long Short-Term Memory (BiLSTM) network twins that take two gesture input samples. The network minimizes a pairwise loss involving a metric distance and similarity label. Unfortunately, *n*-shot learning is not robust against feature distribution shift. Therefore, the probability distributions over two domains, assuming a source and target domain, are brought closer together by minimizing an empirical estimation of the Maximum Mean Discrepancy (MMD) between distributions in an RKHS. Samples are brought into the RKHS automatically when used together with a set of selected kernels through a phenomenon called the ‘kernel trick’.

Chen et al. [9] presented adaptation as a joint classification-reconstruction task. During training, the network performs a classification task and input sample reconstruction task in parallel. The network uses unlabeled samples in the reconstruction task, minimizing the Euclidean distance between an input sample and its reconstruction. Similarly, Yang et al. improved central data aggregation communication efficiency by extracting local intermediate representations at the edge and representation compression by means of quantization [46]. Soltanaghaei et al. [47] and Fang et al. [48] suggested that a neural network can be trained with data containing a combination of domain factors. After model deployment inside a new household, users are occasionally asked to label data samples obtained during critical time periods (i.e., a limited number of periods across time algorithmically determined with large environment occupancy transitions noted in the Wi-Fi–CSI signal) and the neural network is fine-tuned via transfer learning. In addition to transfer learning, Bu et al. [49] added triplet loss based on similar/dissimilar class examples in comparison to an anchor example to increase inter-class distance and decrease the intra-class distance. Shi et al. [50] considered a matching network [51] to learn mapping a small, labeled source support set and an unlabeled target set example to a label, therefore removing the need for specific target domain fine-tuning.

### 6.2. Domain-Independent Learning

Domain-independent learning generally involves either steering model training such that priority is given to feature commonness across domain factors [11] or inclusion of attention mechanisms into a deep neural network. Most steering model training approaches [11,52,53,54,55,56,57,58,59] rely on the availability of domain labels. Often, during network training, as well as having a task output that produces class predictions, the network also has a ’discriminator’ output that produces domain predictions. An adversarial training procedure is used where both outputs play a min-max game. Jiang et al. [11] proposed a balance regularization technique, which adds loss based on Jensen–Shannon divergence [60] between the output probability distribution and an auxiliary distribution derived thereof (for every class, the output probability is normalized by the total number of class predictions with a similar domain label compared to the input domain label). Cui et al. [59] considered pre-training a location predictor first prior to playing a min-max game between a source and target domain discriminator.

Attention refers to putting more cognitive attention toward parts of an input sample that matter for the learning task at hand. The authors of [12] used two attention modules consisting of a sub-neural network. The authors of [61] also used a two attention modules network; however, their novelty was that they combined temporal and frequency attention mechanisms to identify and classify human activities accurately. Temporal attention aims to capture the temporal dynamics of human activities, while frequency attention focuses on extracting discriminative features from the Wi-Fi signal’s frequency domain. Chen et al. [10] created an attention-based BLSTM that is employed to learn the correct representative features in two directions from the raw CSI data. Xu et al. [62] made AGait, for human gait recognition and walking direction estimation. An RNN encoder initially reads and encodes the pre-processed CSI data into primary feature vectors. Then, an attention vector is computed by the decoder and used to predict the label. Hong et al. [63] considered two attention modules, facilitating feature extraction and gesture classification, applied directly to the reshaped intermediate representation. These modules are implemented on the width/height and channel dimensions to retrain learned features correlated to their position and channel. Hu et al. [14] presented *n*-shot-based gesture classification using a backbone network consisting of MobileNet convolutional layer blocks that include a squeeze-and-excitation block (also typically referred to as ’channel-attention’). Yang et al. [64] encoded geometric information in a deep neural network by means of geometric embeddings. This information is encoded as prior domain factor knowledge, therefore functioning as extra inductive bias.

#### Use of Cross-Domain Input Features

The idea of using cross-domain features is to develop a manual algorithm or system of equations based on physics and signal processing expert knowledge. The goal of this algorithm/system is to convert domain-dependent features (e.g., raw CSI or pre-processed input data) into less domain-dependent ones. Zheng et al. [13] created a gesture-recognition system called Widar3. From the CSI data, they first created a Doppler Frequency Shift profile (DFS), which was used to create a body-coordinate velocity profile (BVP). BVP is a less domain-dependent input feature and relies on the power distribution over physical velocity in a person’s body-coordinate system. Zhang et al. [65] introduced a coefficient matrix and normalization procedure extension to BVP to make it applicable to gait recognition involving location variation. Li et al. [35] extracted a Motion Change Pattern (MCP) from a DFS spectrogram. MCP is defined as the derivative of magnitude across time for a specific Doppler frequency. Derivative relevance across Doppler frequencies is represented by a weight matrix that is updated via gradients computed using a convolution operation together with the Sobel [66] operator. MCP was observed to be domain-independent for domain factors involving the same user (e.g., position) during visual MCP inspection. However, differences in start/end times of a gesture’s sub-motions, i.e., the steps taken to fully complete an action (e.g., drawing a line from left to right when drawing a rectangle) across users, make this feature user-dependent. Lu et al. [67] indicated that domain shifts caused by position differences lead to both changes in a signal’s subcarrier attenuation variation (a.k.a. fading) and a signal’s waveform. To mitigate the effect of subcarrier attenuation variation, adaptive subcarrier selection was applied to the signal based on summed entropy across time. To mitigate waveform changes, the authors proposed separating CSI into a low-rank (containing background information) and sparse (containing gesture information) tensor. The low-rank tensor was subsequently ignored. Chen et al. [68] introduced a method for binary motion detection independent of the environment. They proposed a thresholding procedure that involves reshaping CSI magnitude tensors into a matrix format, where each row represents a unique combination of transmitter and receiver sub-carriers, and each column represents a specific time sequence. A variance vector is generated by calculating the variance of elements within each row. This process is repeated multiple times, resulting in a matrix of stacked variance vectors. To analyze the data further, a Variance of Variance (VOV) vector is computed by calculating the variance per row. The thresholding step involves comparing the minimum value of a sliding window of VOV with a predetermined threshold that remains static throughout the process. Gao et al. [69] proposed Motion Navigation Primitive (MNP), a phase-difference ratio between phase-difference signals (signals denoting CSI phasor element’s phase rate of change) originating from two different Wi-Fi transceiver pairs. Phase-difference signals are the derivatives of CSI phase signals. The intuition behind MNP is that it shifts the input data’s perspective from the relative position between the object to be sensed and the transceiver to the relative position between object positions across two timestamps, therefore losing dependence on position and orientation. Dang et al. [70] subtracted CSI antenna phases of reference measurements acquired during an offline measurement period from inference measurements to mitigate localization environmental effects.

## 7. Conclusion and Future Work

In this paper, we validated the hypothesis that ‘*in deep-learning-based Wi-Fi–CSI sensing, domain factor independency, and adaptation limitations can be absolved by steering a feature-extraction model’s training process such that the ability to separate intermediate feature PDFs of input data batches seen previously from a current input data batch PDF artificially permuted with a diffeomorphism is lost*’. Our extensive evaluations of the Widar3 and SignFi gesture-recognition benchmark datasets, while both considering DFS and GADF as input types, showed that this hypothesis is invalid. We identified several pitfalls that may have led to this invalidity, including a lack of good-quality benchmark datasets, a lack of precise descriptions of methodologies, invalid probability distribution assumptions, and problems with non-linear scaling. We also observed that the involvement of newer feature extractor blocks, such as EfficientNet blocks involving a squeeze-and-excitation component, can increase one-orientation leave-out cross-validation performance over older blocks, such as standard CNN and ResNet blocks.

Apart from the user, sensor device placement, environment, and user placement with respect to the sensor device, not much is known about the effect of other factors inducing domain shifts between data used during training or inference, especially in large-scale industrial or social environments. In this regard, future work should be focused on acquiring higher-quality datasets, at least in accordance with the quality level set by the Widar3 dataset, which includes a greater variety of domain-shift-inducing factors. New experiments involving an updated version of the mini-batch alignment pipeline should be created. Updates that should at least be considered include (i) random sampling operation reparametrization, (ii) modeling feature PDF interdependencies, and (iii) making the diffeomorphism-picking process more deterministic regarding achieving a good domain-independent feature representation level.

In addition, we observed that the involvement of adversarial domain classification or involvement of input and intermediate representation attention blocks could achieve better domain factor leave-out performance over a baseline, not considering domain-shift mitigation in different experiment scenarios. Unfortunately, models that use domain labels for learning the extraction of domain-independent features do not scale well to increases in domains because they require new discriminator construction. Future work in this scenario should involve developing deep neural network modules that learn to extract domain-independent features without a domain label or by auto-generating a domain label. Additional experiments involving different attention blocks (e.g., attention in multi-task learning, involvement of transformer-based feature extractors, etc.) should also be considered. Since attention involves making updates to the architecture while adversarial domain classification involves making updates to the way the neural network is trained, their ability to be complementary to each other, therefore boosting the level of achieved domain-invariant feature-extraction capabilities, should also be tested.

Lastly, we noticed that data-collection setup and pre-processing pipeline building-block choices that work for one dataset may not always work on another dataset, causing under-/overfitting on an in-domain validation subset. Under-/overfitting, in turn, overshadows potential domain-shift effects. Therefore, research should be carried out to (i) identify pre-processing methods that generalize well to a multitude of datasets and (ii) how processing methods meant for computation/memory complexity reduction can be integrated with domain-shift mitigation methods without causing severe performance degradation in situations where training and inference data have a domain shift regarding each other.

## Figures and Tables

**Table 1:** Feature extractor with DFS as input type hyperparameter table.

I	Op	KP	It	Ot	E	Ac	St	Se	Sk
128 × 256 × 1	Conv.	5 × 9	-	800	-	Swish	2 × 8	-	-
64 × 32 × 800	MobileV2	4 × 4	8	32	3	Swish	2 × 2	0.15	-
32 × 16 × 32	MobileV2	4 × 4	32	32	3	Swish	1 × 1	0.15	🗸
32 × 16 × 32	MobileV2	4 × 4	80	176	4	Swish	2 × 2	0.1	-
16 × 8 × 176	MobileV2	4 × 4	176	176	4	Swish	2 × 2	0.1	-
8 × 4 × 176	MobileV2	4 × 4	176	176	4	Swish	2 × 2	0.1	-
4 × 2 × 176	Conv.	6 × 7	-	560	-	Swish	2 × 2	-	-
2 × 1 × 560	Max. Pool	2 × 1	-	-	-	-	2 × 1	-	-

Table denotes input dimension (I), operator type (Op), kernel/pool size (KP), nr. output filters used by the first convolution layer inside MobileV2 block (It), nr. output filters (Ot), expansion rate (E), activation function type (Ac), stride (St), if squeeze and excitation is considered, including its rate (Se), and if a skip connection (Sk) is considered. The time dimension size is 2048 for the Widar3 subset and 256 for the SignFi dataset. The transceiver link dimension size is 6 for the Widar3 subset and 1 for the SignFi dataset. The table above only uses the time dimension and transceiver link size for the SignFi dataset.

**Table 2:** Feature extractor with GADF as input type hyperparameter table.

I	Op	KP	It	Ot	E	Ac	St	Se	Sk
256 × 256 × 1	Conv.	26 × 14	-	20	-	Swish	1 × 1	-	-
256 × 256 × 20	Max. Pool	28 × 38	-	-	-	-	2 × 2	-	-
128 × 128 × 20	MobileV2	6 × 6	20	40	4	Swish	1 × 1	0.4	-
128 × 128 × 40	Max. Pool	22 × 22	-	-	-	-	2 × 2	-	-
64 × 64 × 40	MobileV2	3 × 3	40	80	7	Swish	1 × 1	0.7	-
64 × 64 × 80	Max. Pool	40 × 14	-	-	-	-	2 × 2	-	-
32 × 32 × 80	Conv.	6 × 12	-	160	-	Swish	1 × 1	-	-
32 × 32 × 160	Max. Pool	32 × 32	-	-	-	-	32 × 32	-	-

Table denotes input dimension (I), operator type (Op), kernel/pool size (KP), nr. output filters used by the first conv. layer inside MobileV2 block (It), nr. output filters (Ot), expansion rate (E), activation function type (Ac), stride (St), if squeeze and excitation is considered, including its rate (Se), and if a skip connection (Sk) is considered. Timeframe size is 512 for the Widar3 subset and 256 for the SignFi dataset. The transceiver link antenna size is 6 for the Widar3 subset and 1 for the SignFi dataset. The above-listed table only uses the timeframe and transceiver link size for the SignFi dataset.

**Table 3:** JCR decoder with DFS as reconstructed input type hyperparameter table (for SignFi dataset).

I	Op	KP	It	Ot	E	Ac	St	Se	Sk
2 × 1 × 560	Conv.	6 × 7	-	560	-	Swish	1 × 1	-	-
2 × 1 × 560	MobileV2	4 × 4	176	176	4	Swish	2 × 2	0.1	-
4 × 2 × 176	MobileV2	4 × 4	176	176	4	Swish	2 × 2	0.1	-
8 × 4 × 176	MobileV2	4 × 4	176	80	4	Swish	2 × 2	0.1	-
16 × 8 × 80	MobileV2	4 × 4	32	32	3	Swish	1 × 1	0.15	🗸
16 × 8 × 32	MobileV2	4 × 4	32	8	3	Swish	2 × 2	0.15	-
32 × 16 × 8	Upsample	-	-	-	-	-	2 × 2	-	-
64 × 32 × 8	Conv.	5 × 9	-	800	-	Swish	1 × 1	-	-
64 × 32 × 800	Conv. Trans.	5 × 9	-	1	-	Swish	2 × 8	-	-

Table denotes input dimension (I), operator type (Op), kernel/pool size (KP), nr. output filters used by the first conv. layer inside MobileV2 block (It), nr. output filters (Ot), expansion rate (E), activation function type (Ac), upsampling rate (Ur), if squeeze and excitation is considered, including its rate (Se), and if a skip connection (Sk) is considered. The time dimension size is 2048 for the Widar3 subset and 256 for the SignFi dataset. The transceiver link dimension size is 6 for the Widar3 subset and 1 for the SignFi dataset. The table above only uses the time dimension and transceiver link size for the SignFi dataset.

**Table 4:** JCR decoder with GADF as reconstructed input type hyperparameter table (for SignFi dataset).

I	Op	KP	It	Ot	E	Ac	St	Se	Sk
32 × 32 × 160	MobileV2	3 × 3	80	40	7	Swish	1 × 1	0.7	-
32 × 32 × 40	Upsample	-	-	-	-	-	2 × 2	-	-
64 × 64 × 40	MobileV2	6 × 6	40	20	4	Swish	1 × 1	0.4	-
64 × 64 × 20	Upsample	-	-	-	-	-	2 × 2	-	-
128 × 128 × 20	Conv.	14 × 30	-	20	-	Swish	1 × 1	-	-
128 × 128 × 20	Upsample	-	-	-	-	-	2 × 2	-	-
256 × 256 × 20	Conv.	42 × 12	-	1	-	Swish	1 × 1	-	-

Table denotes input dimension (I), operator type (Op), kernel/pool size (KP), nr. output filters used by the first conv. layer inside MobileV2 block (It), nr. output filters (Ot), expansion rate (E), activation function type (Ac), upsampling rate (Ur), if squeeze and excitation is considered, including its rate (Se), and if a skip connection (Sk) is considered. Timeframe size is 512 for the Widar3 subset and 256 for the SignFi dataset. The transceiver link antenna size is 6 for the Widar3 subset and 1 for the SignFi dataset. The table above only uses the timeframe and transceiver link size for the SignFi dataset.

**Table 5:** ATT input attention block hyperparameter table (for SignFi dataset).

I	Op	KP	It	Ot	E	Ac	St	Se	Sk
128 × 256 × 1	Conv.	48 × 32	-	1	-	Sigmoid	1 × 1	🗸	-

Table denotes input dimension (I), operator type (Op), kernel/pool size (KP), nr. output filters used by the first conv. layer inside MobileV2 block (It), nr. output filters (Ot), expansion rate (E), activation function type (Ac), stride (St), if batch normalization is considered (Bn), and if a skip connection (Sk) is considered. The frame size is 2048 or 512 for the Widar3 subset when considering DFS or GADF as input type and 256 for the SignFi dataset. The transceiver link antenna size is 6 for the Widar3 subset and 1 for the SignFi dataset.

**Table 6:** ATT latent representation attention block hyperparameter tables (for the SignFi dataset).

I	Op	KP	It	Ot	E	Ac	St	Se	Sk
2 × 1 × 560	Max. Pool	28 × 14	-	1	-	-	22 × 26	-	-
1 × 1 × 560	Shared MLP	-	-	-	-	-	-	-	-
(**a**) F1									
**I**	**Op**	**KP**	**It**	**Ot**	**E**	**Ac**	**St**	**Se**	**Sk**
2 × 1 × 560	Avg. Pool	22 × 20	-	-	-	-	22 × 26	-	-
1 × 1 × 560	Shared MLP	-	-	-	-	-	-	-	-
(**b**) F2									
**I**	**Op**	**KP**	**It**	**Ot**	**E**	**Ac**	**St**	**Se**	**Sk**
1 × 1 × 560	Flatten	-	-	-	-	-	-	-	-
560	Dense	-	-	1184	-	ReLU	-	-	-
1184	Dense	-	-	624	-	ReLU	-	-	-
624	Dense	-	-	560	-	ReLU	-	-	-
560	Reshape	-	-	-	-	-	-	-	-
(**c**) Shared MLP									

Tables denote input dimension (I), operator type (Op), kernel/pool size (KP), nr. output filters used by the first conv. layer inside MobileV2 block (It), nr. output filters (Ot), expansion rate (E), activation function type (Ac), stride (St), if batch normalization is considered (Bn), and if skip connection (Sk) is considered.

**Table 7:** Widar3 DFS One-domain-factor leave-out cross-validation result comparison summary.

User	STD	ADV	ATT	JCR	MBA
STD	.	x	🗸	🗸	-
ADV	🗸	.	🗸	🗸	🗸
ATT	x	x	.	x	x
JCR	x	x	🗸	.	-
MBA	-	x	🗸	-	.
**Position**	**STD**	**ADV**	**ATT**	**JCR**	**MBA**
STD	.	🗸	x	x	x
ADV	x	.	x	x	x
ATT	🗸	🗸	.	x	x
JCR	🗸	🗸	🗸	.	-
MBA	🗸	🗸	🗸	-	.
**Orientation**	**STD**	**ADV**	**ATT**	**JCR**	**MBA**
STD	.	x	x	x	x
ADV	🗸	.	🗸	🗸	-
ATT	🗸	x	.	🗸	x
JCR	🗸	x	x	.	x
MBA	🗸	-	🗸	🗸	.

Symbols: (.) irrelevant, (🗸) row technique > column technique, (-) no noticeable difference, (x) row technique < column technique. Row with the most 🗸 symbols can be considered the best-performing domain-shift mitigation technique.

**Table 8:** Widar3 DFS two-orientation-factor leave-out cross-validation result comparison summary.

Orientation	STD	ADV	ATT	JCR	MBA
STD	.	🗸	x	🗸	🗸
ADV	x	.	x	🗸	-
ATT	🗸	🗸	.	🗸	🗸
JCR	x	x	x	.	x
MBA	x	-	x	🗸	.

Symbols: (.) irrelevant, (🗸) row technique > column technique, (-) no noticeable difference, (x) row technique < column technique. Row with the most 🗸 symbols can be considered the best-performing domain-shift mitigation technique.

## Data Availability

The Widar3 dataset can be accessed via IEEE DataPort [accessed on 20 May 2022] (https://ieee-dataport.org/open-access/widar-30-wifi-based-activity-recognition-dataset). Links to the SignFi dataset can be found at Github page [accessed on 20 May 2022] (https://github.com/yongsen/SignFi).
